# Sarcoptic mange severity is associated with reduced genomic variation and evidence of selection in Yellowstone National Park wolves (*Canis lupus*)

**DOI:** 10.1111/eva.13127

**Published:** 2020-09-20

**Authors:** Alexandra L. DeCandia, Edward C. Schrom, Ellen E. Brandell, Daniel R. Stahler, Bridgett M. vonHoldt

**Affiliations:** ^1^ Ecology & Evolutionary Biology Princeton University Princeton NJ USA; ^2^ Biology Pennsylvania State University State College PA USA; ^3^ Yellowstone Center for Resources Yellowstone National Park WY USA

**Keywords:** ectoparasite, genetics, infection severity, mite infestations, natural selection, RAD‐sequencing, sarcoptic mange, wildlife disease

## Abstract

Population genetic theory posits that molecular variation buffers against disease risk. Although this “monoculture effect” is well supported in agricultural settings, its applicability to wildlife populations remains in question. In the present study, we examined the genomics underlying individual‐level disease severity and population‐level consequences of sarcoptic mange infection in a wild population of canids. Using gray wolves (*Canis lupus*) reintroduced to Yellowstone National Park (YNP) as our focal system, we leveraged 25 years of observational data and biobanked blood and tissue to genotype 76,859 loci in over 400 wolves. At the individual level, we reported an inverse relationship between host genomic variation and infection severity. We additionally identified 410 loci significantly associated with mange severity, with annotations related to inflammation, immunity, and skin barrier integrity and disorders. We contextualized results within environmental, demographic, and behavioral variables, and confirmed that genetic variation was predictive of infection severity. At the population level, we reported decreased genome‐wide variation since the initial gray wolf reintroduction event and identified evidence of selection acting against alleles associated with mange infection severity. We concluded that genomic variation plays an important role in disease severity in YNP wolves. This role scales from individual to population levels, and includes patterns of genome‐wide variation in support of the monoculture effect and specific loci associated with the complex mange phenotype. Results yielded system‐specific insights, while also highlighting the relevance of genomic analyses to wildlife disease ecology, evolution, and conservation.

## 1 INTRODUCTION

A classic paradigm in population genetics states that molecular diversity buffers against disease risk (Spielman, Brook, Briscoe, & Frankham, 2004). Host variation is thought to confer multiple defense strategies, thus limiting a pathogen's ability to exploit common weaknesses at the individual and population levels (Bergstrom & Antia, 2006; Hedrick, 1999). Conversely, the absence of host variation is expected to increase a population's vulnerability to infection, leading to disease outbreaks. This phenomenon is well supported within agricultural settings (Reiss & Drinkwater, 2018) and has been termed the “monoculture effect” (Elton, 1958). However, the universality of this trend beyond the agricultural realm remains uncertain.

Elucidating the relationship between host genomic variation and wildlife disease remains a prominent goal of molecular and disease ecologies (Blanchong, Robinson, Samuel, & Foster, 2016; DeCandia, Dobson, & vonHoldt, 2018). This is particularly important for small, fragmented, or reintroduced populations, where genetic diversity loss may reduce evolutionary potential and threaten long‐term viability (Frankham, 2005; Spielman, Brook, & Frankham, 2004). Regarding disease, the inability to cope with novel or enduring parasites can lead to increased morbidity among individuals, and ultimately precipitate population declines or local extirpation. This phenomenon remains understudied in wild populations, with little consensus between disparate host–parasite systems.

A recent meta‐analysis found strong support for the monoculture effect in wildlife by examining the effect of population‐level heterozygosity on parasite success (Ekroth, Rafaluk‐Mohr, & King, 2019). However, this study primarily focused on invertebrate hosts and included both laboratory‐ and field‐based studies. Its focus on population‐level heterozygosity further excluded consideration of individual‐level effects. Although relatively few in number, within‐population studies in wildlife have reported an inverse relationship between genetic diversity and disease using neutral microsatellites (Coltman, Pilkington, Smith, & Pemperton, 1999; Townsend et al., 2018), immunogenetic markers (Brambilla, Keller, Bassano, & Grossen, 2018), and genome‐wide datasets (Banks et al., 2020). In addition, morbidity has been associated with specific loci in multiple host species (Batley et al., 2019; Donaldson et al., 2017; Elbers, Brown, & Taylor, 2018; Ellison et al., 2014; Margres et al., 2018). Considered together, these studies highlight the importance of characterizing genetic variation within the context of wildlife disease, particularly for conservation‐relevant species.

We contributed to these efforts by examining host genomic variation and infection severity in a wild population of canids: gray wolves (*Canis lupus*) inhabiting Yellowstone National Park (YNP). YNP wolves have been closely monitored for disease since their initial reintroduction in 1995 and 1996 (Phillips & Smith, 1997). To minimize risk, founders were screened for good health, vaccinated against numerous canine diseases, and treated with a broad‐spectrum acaricide and anthelmintic (Almberg, Cross, Dobson, Smith, & Hudson, 2012). As a result, founders and their offspring initially bore low disease loads. Within a few generations, however, their light burden gave way to high exposure of canine adenovirus type‐1, canine parvovirus, canine herpesvirus, and the protozoan *Neospora caninum* (Almberg, Mech, Smith, Sheldon, & Crabtree, 2009). These diseases were considered enzootic in the park's canids, and none appeared to negatively impact individual fitness or population viability.

**FIGURE 1:**
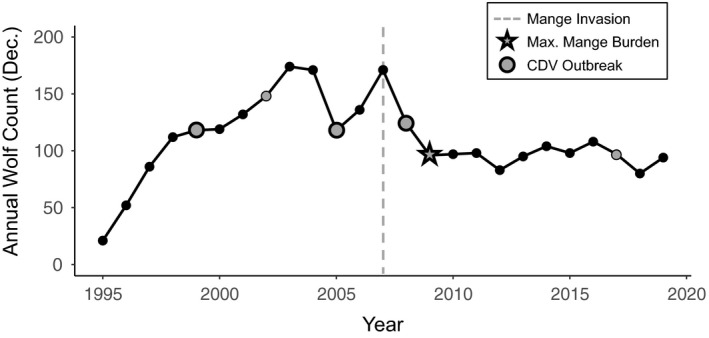
Annual wolf counts recorded in December 1995 through 2019 with years of CDV outbreaks, mange invasion, and maximum mange burden indicated (figure adapted from Almberg et al., 2012). Large circles represent large‐scale CDV outbreaks, with smaller circles indicative of smaller outbreaks

Conversely, canine distemper virus (CDV) and sarcoptic mange have been associated with morbidity, mortality, and reduced population size in YNP (Figure 1; Almberg et al., 2012). Large‐scale outbreaks of CDV in 1999, 2005, and 2008 (with smaller outbreaks in 2002 and 2017) infected multiple carnivore hosts in the Greater Yellowstone Ecosystem, leading to high levels of wolf‐pup mortality (Almberg et al., 2009, 2011; Almberg, Cross, & Smith, 2010; Stahler, Macnulty, Wayne, vonHoldt, & Smith, 2013). While the effects of most outbreaks were short‐lived, the 2008 outbreak coincided with the invasion of sarcoptic mange in YNP wolves. Combined with density‐dependent mortality caused by interpack aggression (Cubaynes et al., 2014), the 2008 CDV outbreak and 2007 mange invasion appeared to have regulated population size, which has stabilized between 80 and 108 wolves since (Almberg et al., 2012; Smith et al., 2020).

Sarcoptic mange is caused by the ectoparasitic mite *Sarcoptes scabiei* and has been observed in YNP wolves every year since its invasion in January 2007 (Almberg et al., 2012, 2015; Pence & Ueckermann, 2002). Symptoms include pruritus, alopecia, eosinophilia, hyperkeratosis, hyperpigmentation, and dermal inflammation (Almberg et al., 2012; Bornstein, Morner, & Samuel, 2001; Nimmervoll et al., 2013; Oleaga, Casais, Prieto, Gortázar, & Balseiro, 2012). These symptoms are consistent with type IV (or delayed) hypersensitivity, which suggests that an ineffective immune response harms the host through chronic inflammation (Abbas, Lichtman, & Pillai, 2016). Yet, the severity of these symptoms varies widely among wolves. Some individuals develop minor symptoms, rapidly clear mites, and fully recover within months. Others quickly develop severe symptoms that worsen until death from mange or its associated dehydration, emaciation, secondary bacterial infection, or increased vulnerability to other causes such as intraspecific killings (Almberg et al., 2012; DeCandia, Leverett, & vonHoldt, 2019). As the source of this variability remains unknown, mange is considered a ubiquitous yet neglected disease (Hengge, Currie, Jäger, Lupi, & Schwartz, 2006; Walton, Holt, Currie, & Kemp, 2011).

We hypothesized that host genomic variation contributes to differences in mange infection severity in YNP wolves. More specifically, we predicted that infection severity would inversely correlate with genome‐wide diversity. Through implementation of a family‐based association study, we anticipated identification of associated loci with putative gene functions related to immunity, inflammation, and skin barrier integrity, highlighting the relevance of specific variants to the mange phenotype. As numerous factors are known to contribute to disease state in wildlife, we predicted that genetic variation would be one of several variables predictive of mange severity at the individual level, with environmental and pack‐level variables also relevant. We further considered changes in genomic variation through time at the population level. Here, we anticipated reductions of genome‐wide variation since the initial reintroduction events in 1995–1996, as YNP typically serves as a source population for surrounding areas rather than a sink for dispersers (vonHoldt et al., 2010). We additionally predicted that mange‐associated alleles would reduce in frequency following the 2007 invasion of mange, as we hypothesized that severe infection exerts selective pressure on YNP wolves.

To test these hypotheses, we generated a genome‐wide dataset of single nucleotide polymorphisms (SNPs) in a subset of YNP wolves exposed to sarcoptic mange for individual‐level analyses. We then genotyped these same SNPs in all wolves with biobanked blood and tissue for population‐level analyses. The availability of samples and detailed phenotypic data for YNP wolves uniquely enabled us to disentangle genetic and environmental factors underlying this complex disease phenotype. As mange infects over one hundred mammal species worldwide including humans (Pence & Ueckermann, 2002), YNP wolves serve as a case study with important implications for mammals globally. This study advances our understanding of the genomics underlying mange in a free‐ranging carnivore, while also providing insights and predictions applicable to diverse host–parasite systems.

## 2 METHODS

### 2.1 Study area

YNP encompasses 8,991 km^2^ of protected land in north‐western Wyoming and adjacent parts of Montana and Idaho in the western United States. YNP is mountainous (elevation range: 1,500–3,800 m), and its steep gradients in elevation, soil, and climate contribute to varied land cover, including riparian vegetation, shrubland, grassland, alpine meadows, and mixed coniferous forests. We make reference to two regions of the park, the northern range and the interior, based on ecological and physiographical differences and variation in disease dynamics (see Almberg et al., 2009, 2012 for details). Importantly, the 1,000 km^2^ area of the northern range within YNP is characterized by lower elevations (1,500–2,200 m), serves as prime wintering habitat for the park's ungulates, and supports a higher density of wolves than the interior. In contrast, the interior (7,991 km^2^) is higher in elevation (>2,500 m), receives higher annual snowfall, and generally supports lower densities of wolves and ungulates.

### 2.2 Sample collection and mange classification

We used archived tissue and blood samples collected by the National Park Service (NPS) during field necropsies and annual helicopter capture and handling of YNP wolves conducted in accordance with NPS Institutional Animal Care and Use Committee (IACUC permit IMR_YELL_Smith_wolves_2012). Sample collection procedures were also reviewed and approved by the Princeton University Institutional Animal Care and Use Committee (Princeton IACUC #2009A‐17).

Annually, both static and dynamic life history data were collected on YNP wolves. Static metadata included sex, coat color (gray or black), date of birth, natal pack, and date of death. Dynamic metadata included annual records of pack membership, age group, and social status. In cases where sex was undetermined (i.e., decomposed carcasses), we used a simple molecular assay of sex chromosomes to infer sex (DeCandia, Gaughran, Caragiulo, & Amato, 2016). Pack‐level variables included location in the park (northern range or interior), pack size, and breeding status (i.e., whether the pack contained a breeding pair that year).

Frequent observations of YNP wolves also resulted in the documentation of individual mange scores, which reflected the percentage of body area presenting symptoms, such as hair loss or lesions. On a 3‐point scale, a score of 0 indicated no evidence of mange, 1 indicated that ≤ 5% of the body was impacted by mange‐related symptoms, 2 referred to 6%–50% of the body being symptomatic, and 3 referred to the most severe score where > 50% of the body was presenting symptoms (Almberg et al., 2012; Pence, Windberg, & Sprowls, 1983). Any field‐based observation or annual capturing of animals by NPS resulted in a mange score assigned to the corresponding individual. The frequent monitoring by NPS officials, consistent method of mange score assignment, and repeated observation of the same wolves maximized confidence in disease phenotypes. In downstream statistical analyses, we used the highest mange score documented per wolf for genetic analyses and mange score at the time of observation for mixed‐effects modeling. Severity classes were coded “mild” for highest score 1, “moderate” for highest score 2, and “severe” for highest score 3.

To estimate pack‐level exposure, we used the dates of first and last mange observation for each pack. We then flanked these dates by one month to account for asymptomatic periods that can both precede and follow infection (Arlian, 1989; Samuel, 1981). This established the mange exposure window for each member in the pack. We restricted our mange dataset to only include wolves that contained three or more observations and were putatively exposed to mange, even if the animal was assigned a mange score of 0 (following vonHoldt et al., 2020).

### 2.3 DNA extraction and restriction site‐associated DNA (RAD) sequencing

We extracted genomic DNA following the Qiagen DNeasy Blood & Tissue Kit standard protocol, quantified samples with the Quant‐iT™ PicoGreen® dsDNA Assay Kit or Qubit^TM^ fluorometric quantitation, and standardized concentrations to 5 ng/μL. We additionally visualized DNA extractions on 1% agarose gels to identify and retain samples with high molecular weight for library preparation.

We used a modified restriction site‐associated DNA sequencing (RADseq) protocol by Ali et al. (2016) to generate genome‐wide SNP data. To summarize, we digested genomic DNA with the *sbfI* restriction enzyme prior to ligation of uniquely barcoded, biotinylated adaptors. We then pooled barcoded samples (48 samples per pool) and randomly sheared DNA to 400 bp on a Covaris LE220. Sheared libraries were enriched for fragments containing the ligated adaptor using a streptavidin bead‐binding assay (Dynabeads M‐280, Invitrogen), with subsequent library preparation following the standard manufacturer protocol for the NEBNext Ultra II DNA Library Preparation Kit (New England Biolabs). We purified and selected libraries for fragments 300–400 bp in size using Agencourt AMPure XP magnetic beads. Two libraries were pooled for each final sequencing library to contain 96 barcoded samples. Final libraries were then standardized to 10 nM before paired‐end sequencing (2X150 nt) on an Illumina HiSeq 2500 or NovaSeq 6000.

We used a custom perl script (sbfI_flip_trim_150821.pl, see Appendix S1) to align all forward and reverse reads with the restriction enzyme cut site into one file. We then used *STACKS v1.42* (Catchen, Hohenlohe, Bassham, Amores, & Cresko, 2013) for the initial stages of data processing, in order to manually remove poor‐quality samples from the dataset before paired‐end mapping. We used *process_radtags* to demultiplex and filter reads for > 2bp barcode mismatches or quality scores below 90% using a sliding window (15% of the read), and removed PCR duplicates using default parameters in *clone_filter*.

We completed paired‐end alignments to the reference dog CanFam3.1 genome (Lindblad‐Toh et al., 2005) using *STAMPY v1.0.21* (Lunter and Goodson 2011) for samples with > 500,000 reads to maximize sequence coverage. SAM files were sorted and filtered for quality scores (MAPQ) ≥ 96 using *Samtools v0.1.18* (Li et al., 2009), with a final conversion to BAM format. We subsequently used *STACKS v2.2* to identify, genotype, and filter SNPs with *gstacks* and *populations* for paired‐end data using the Marukilow model (Maruki & Lynch, 2017). As this model assesses the statistical likelihood of each genotype call, it reduces the need for subsequent coverage filters when paired with clone‐filtering.

We implemented the *populations* module using all available samples and the flag–write_single_snp to retain only a single polymorphic site per read. When samples were replicated in the library preparation and sequencing process, we used *PLINK* (Purcell et al., 2007) to compare each of the replicates for proportion of missing loci and retained the sample with lower missingness. We excluded wolves with fewer than three observations and no history of mange exposure (based on pack membership and infection history), as it was impossible to assess mange severity class in individuals never challenged by mites. To complete the dataset with this final set of samples, we implemented the *STACKS v2.2 populations* module a second time with an additional filtering parameter (−*r* 0.9) to retain loci genotyped in more than 90% of wolves. We used *VCFtools v0.1.12b* (Danecek et al., 2011) to remove singletons, doubletons, and sites found on the X chromosome, due to difficulties posed by chromosomal sex determination and X‐inactivation to mixed‐sex study designs (Clayton, 2009). This produced our final dataset of high‐confidence autosomal SNPs for downstream analysis. For population‐level analyses, we genotyped these same SNPs in all wolves (regardless of mange exposure history) with available biomaterial.

### 2.4 Genetic diversity statistics

We hypothesized that genetic diversity would inversely correlate with mange infection severity, as coded into classes mild, moderate, and severe. We used *STACKS v2.2* to examine patterns of genetic diversity between: (a) infected and uninfected wolves and (b) infected wolves with different mange severities. Diversity metrics included the percentage of polymorphic sites (%Poly), number of private alleles (PAS), minor allele frequency (MAF), observed heterozygosity (*H_O_*), expected heterozygosity (*H_E_*), and nucleotide diversity (*π*). We assessed the statistical significance of between‐group differences in *R*. For binary mange presence, we used two‐tailed Welch's *t* tests, as we assumed unequal variance between the two infection groups. For infection severity, we used analysis of variance (ANOVA), as this allowed for inclusion of all four mange severity groups in the same analysis.

We next used *ADZE v1.0* to estimate rarefied metrics of allelic diversity (Szpiech, Jakobsson, & Rosenberg, 2008). As allelic richness (*A_R_*), private allelic richness (*PA* *_R_*), and shared *PA* *_R_* are heavily influenced by sample size, adoption of a rarefaction approach enables cross‐group comparisons when sample sizes differ. Using this approach, *A_R_*_,_ *PA* *_R_*, and shared *PA* *_R_* are estimated by averaging subsamples of each group at standardized sample sizes. We set the missing data tolerance to 25% and calculated mean *A* *_R_*, *PA* *_R_*, and shared *PA* *_R_* between infection severity groups.

### 2.5 Mixed‐effects modeling

We used mixed‐effects modeling to contextualize genetic diversity within the broad range of factors that may influence infection severity in YNP wolves. Input data were derived from annual observations conducted in YNP between 2007 and 2019, and included both static and dynamic life history variables for infected wolves (mange status of 1, 2, or 3). Mange status at the time of observation served as the response variable, and both random and fixed effects were considered during model selection. Individual wolves appeared in the dataset multiple times, with their static life history variables unchanged and their dynamic life history variables sometimes differing.

To control for repeated measures, random‐effects variables included individual identifier, pack membership at the time of observation, and year observed. Individual identifier controlled for nonindependence between repeated measures of the same wolf, whereas pack membership and year observed controlled for shared environmental effects present within each pack (Almberg et al., 2015; Brzeski et al., 2015) and observation year (Stahler et al., 2013). As numerous wolves changed pack membership across years, we fitted these variables as partially crossed random intercepts.

Fixed effects included environmental (season and location in the park), pack‐level (breeding status and size of the pack), and individual‐level (sex, coat color, age group, social status, and standardized observed heterozygosity or *H_O_*) variables. To determine season, we assigned observations obtained during October through March as “winter,” and those obtained during April through September as “summer.” For each observation, we used pack membership to determine location in the park (northern range or interior), estimated pack size (range 1–18), and pack‐level breeding status (yes or no) for that observation year. Sex (male or female), coat color (gray or black), age group (yearling or adult), and social status (subordinate or alpha) were assigned at each observation, with missing data interpolated using adjacent observations and YNP Annual Reports (2007–2019). To account for genetic diversity, we standardized *H_O_* calculated for each wolf by subtracting the mean and dividing by standard deviation. We chose this measure to represent genetic diversity as standardized *H_O_* provided the most inclusive estimate of genome‐wide variation without overparameterizing candidate models. We then formulated a priori hypotheses about how each variable may affect mange infection severity based on existing literature (Table S1; Almberg et al., 2012, 2015; Candille et al., 2007; Cross et al., 2016; DeCandia et al., 2018; Fazal, Cheema, Maqbool, & Manzoor, 2014; Feather, Gough, Flynn, & Elsheikha, 2010; Mech & Boitani, 2003; Oleaga et al., 2011; Pence et al., 1983; Spielman, Brook, Briscoe, et al., 2004; Stahler et al., 2013).

We used cumulative link mixed models (CLMM) implemented in the *R* package *ordinal* v2019.12‐10 to test these hypotheses (Christensen, 2019). A type of generalized linear mixed‐effects model, CLMMs are optimized for ordinal response variables and employ a maximum‐likelihood framework for parameter estimation using the Laplace approximation. We initiated model selection by constructing a null model (no fixed effects) and a global model (all nine fixed effects). We used the saturated model to check for collinearity between fixed effects using the *check_collinearity* function in the *R* package *performance* v0.4.5 (Lüdecke, Makowski, Waggoner, & Patil, 2020). We then implemented a stepwise model reduction procedure, where we sequentially removed the nonsignificant term with the highest *p‐*value in each CLMM (Stahler et al., 2013). We compared sequential models using the likelihood‐ratio test (*lrt*) for cumulative link models, and halted the reduction process when removal of the next fixed effect variable led to significantly worse model fitting. We reconsidered dropped terms by adding them to the reduced model one at a time and implementing *lrt*s to assess significance. We performed a similar procedure for pairwise interaction terms between fixed effects contained in the reduced CLMM to see whether their inclusion significantly improved model fitting. We calculated Akaike information criterion adjusted for small sample (AICc) using *AICcmodavg* v2.2‐2 (Mazerolle, 2019) and weighted AICc using *MuMIn* v1.43.15 (Bartoń, 2019).

### 2.6 Identifying outlier loci

We implemented a univariate linear mixed model in *GEMMA* to identify outlier loci associated with infection severity (Zhou & Stephens, 2012, 2014). We included sex and coat color as covariates in the model to account for static life history variables, and used a pairwise relatedness matrix to account for familial structure within the dataset. As our dataset included wolves with unknown pedigree relationships, we calculated a centered pairwise relatedness matrix using the ‐gk 1 flag in *GEMMA*. We excluded natal pack as a covariate, as the pairwise relatedness matrix accounted for all possible relatives, rather than relying on inferred relations sharing a natal pack. We adjusted the *lrt p*‐values obtained using a modified false discovery rate (FDR) procedure (Benjamini & Yekutieli, 2001), and used an in‐house python script (vonHoldt, Heppenheimer, Petrenko, Croonquist, & Rutledge, 2017) to annotate significant outliers as intronic, exonic, intergenic, or within 2Kb of a promoter in the reference dog genome (Lindblad‐Toh et al., 2005). We predicted functional relevance using the Ensembl Variant Effect Predictor (VEP) web interface (McLaren et al., 2016) and queried genic sites in the Ensembl, Online Mendelian Inheritance in Man (OMIM, 2018), and GeneCards (www.genecards.org) databases. Finally, we used *G:GOST* in *G:PROFILER* to conduct gene ontology analyses (Raudvere et al., 2019). We searched annotated genes for all available annotations (including molecular functions, cellular components, and biological processes) and assessed statistical significance using the Benjamini–Hochberg FDR of 0.05 (Benjamini & Hochberg, 1995; vonHoldt et al., 2020). Although this analysis may be underpowered due to sample size constraints, we adjusted significance thresholds to account for multiple testing and decrease the likelihood of false positives (following DeCandia, Brzeski, et al., 2019; vonHoldt et al., 2020).

### 2.7 Population‐level analyses

We next considered changes in genetic variation through time in all YNP wolves with available biomaterial, regardless of mange exposure history. Using static metadata, dynamic observations, and YNP annual reports, we determined which wolves were alive in each observation year between 1995 and 2019. We then used *ADZE v1.0* to estimate annual mean allelic richness, while controlling for sample size differences between years. For these analyses, we used the missing data tolerance of 100% to ensure that the same loci were analyzed in each year's calculation. To account for the breeding structure of YNP wolves, we performed these analyses a second time using only known breeders. Here, we considered breeding status to be a static life history variable, in order to increase annual sample sizes. As such, each year's calculation included all living breeders regardless of their reproduction status in that particular year.

Following examination of genome‐wide allelic richness, we explored the change in per‐locus allele frequencies through time. For this analysis, we binned loci into three categories based on the association of the focal allele (typically the minor allele) with mange severity in *GEMMA*. Categories included (a) no association, (b) positive association (where increased allele frequency was associated with more severe mange), and (c) negative association (where increased allele frequency was associated with milder mange). We constructed a mixed‐effects model with a Gaussian likelihood and weakly regularizing skeptical priors in the *R* package *brsm* (Bürkner, 2017) to assess whether positive and negative association with mange severity influenced changes in per‐locus allele frequency through time. In this model, standardized allele frequency was regressed on the fixed effects of year, association with mange, and the interaction between them, while controlling for the locus ID of each allele as a random effect. To improve MCMC convergence times, we included all positively and negatively associated alleles but only a randomly selected subsample of 500 nonassociated alleles. For each subset of alleles, the model was run twice: once for the years 1995–2006, and once for the years 2006–2019, to assess changes in allele frequency before versus after the 2007 mange invasion of YNP. The approach was repeated four times to confirm that results were consistent across independent subsamples of nonassociated alleles and across independent Markov chains.

## 3 RESULTS

### 3.1 RAD‐sequencing

Our first implementation of *populations* catalogued 214,762 variant sites in 510 samples. After removing duplicates, wolves with fewer than three observations, and putatively unexposed individuals, we implemented *populations* a second time to create a mange‐relevant dataset with an additional filtering parameter that removed SNPs genotyped in < 90% of individuals. This resulted in 106,936 SNP loci genotyped in 117 wolves. We then filtered SNPs to remove singletons, doubletons, X chromosome sites, and loci missing allelic depth information. The final dataset retained 76,859 SNPs genotyped in 117 wolves, with 49 uninfected and 68 infected individuals. Within the infected group, wolves exhibited mild (*n* = 29, highest mange score 1), moderate (*n* = 26, highest mange score 2), and severe (*n* = 13, highest mange score 3) symptoms (Figure S1). For population‐level analyses, we genotyped these same 76,859 SNPs in 408 unique individuals, regardless of mange exposure history.

### 3.2 Genetic diversity

Regarding susceptibility (i.e., binary mange presence), infected wolves (inf) exhibited higher levels of genetic diversity than wolves with no detected infection (uninf) across several diversity metrics (Table S2). This relationship was statistically significant for observed (two‐sided *t* test; *H_O_*, inf = 0.1986 ± 0.0006, uninf = 0.1942 ± 0.0006, *t*_153650_ = −5.110, *p* < .001) and expected (two‐sided *t* test; *H_E_*, inf = 0.1874 ± 0.0005, uninf = 0.1856 ± 0.0005, *t*_153720_ = −2.367, *p* = .018) heterozygosity, but not for minor allele frequency (two‐sided *t* test; MAF, inf = 0.1249 ± 0.0005, uninf = 0.1239 ± 0.0005, *t*_153710_ = −1.521, *p* = .128) or nucleotide diversity (two‐sided *t* test; *π*, inf = 0.1888 ± 0.0005, uninf = 0.1876 ± 0.0006, *t*_153710_ = −1.623, *p* = .105). Although the number of private alleles was higher in the infected group, sample size differences likely contributed to this result. We therefore used rarefaction to estimate mean allelic richness (*A_R_*) and mean private allelic richness (*PA* *_R_*) across standardized sample sizes in infected and uninfected wolves. We found that uninfected wolves exhibited higher levels of allelic variation across both diversity metrics (*A_R_*, inf = 1.9276 ± 0.0007, uninf = 1.9431 ± 0.0007; *PA* *_R_*, inf = 0.0498 ± 0.0006, uninf = 0.0653 ± 0.0007; Table S3).

**FIGURE 2:**
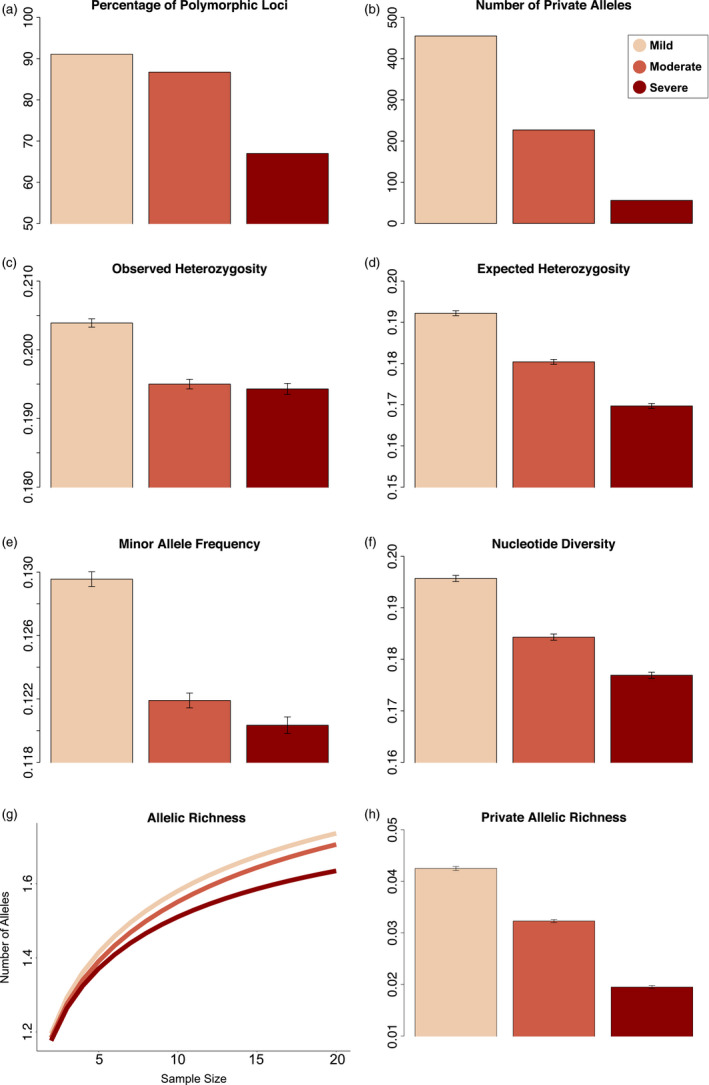
Genetic diversity statistics for mange‐infected wolves grouped by mild (highest mange score 1), moderate (highest mange score 2), and severe (highest mange score 3) infection severity. Metrics include the following: (a) percentage polymorphic loci, (b) number of private alleles, (c) observed heterozygosity, (d) expected heterozygosity, (e) minor allele frequency, (f) nucleotide diversity, (g) rarefied mean allelic richness, and (h) rarefied mean private allelic richness

When grouped by infection severity (uninfected, mild, moderate, and severe), we consistently observed significant differences between severity groups across diversity metrics, including observed (ANOVA; *H_O_*, *F*_1,307434_ = 7.970, *p* = .005) and expected (ANOVA; *H_E_*, *F*_1,307434_ = 558.900, *p* < .001) heterozygosity, minor allele frequency (ANOVA; MAF, *F*_1,307434_ = 73.280, *p* < .001), and nucleotide diversity (ANOVA; π, *F*_1,307434_ = 283.700, *p* < .001). Notably, within the infected groups (mild, moderate, and severe), we observed decreasing genetic diversity with increasing infection severity across all metrics (Figure 2a–f). Uninfected wolves had the largest percentage of polymorphic loci and number of private alleles, but were otherwise intermediate when compared to other severity classes (Table S2; Figure S2).

Similar patterns emerged when controlling for sample size. Mean allelic richness was highest in mildly infected wolves (*A_R_* = 1.7360 ± 0.0011), intermediate in moderately infected wolves (*A_R_* = 1.7059 ± 0.0012), and lowest in severely infected wolves (*A_R_* = 1.6347 ± 0.0016; Figure 2g, Table S3), with nonoverlapping standard errors between all estimates. Regarding mean private allelic richness, mildly infected wolves harbored the most unique alleles (*PA* *_R_* = 0.0425 ± 0.0004), with moderately infected wolves intermediate (*PA* *_R_* = 0.0323 ± 0.0003) and severely infected wolves possessing the fewest (*PA* *_R_* = 0.0195 ± 0.0003; Figure 2h, Table S3). Uninfected wolves exhibited the second highest allelic richness (*A_R_* = 1.7190 ± 0.0011) and possessed the most unique alleles (*PA* *_R_* = 0.0469 ± 0.0004; Table S3; Figure S2).

**FIGURE 3:**
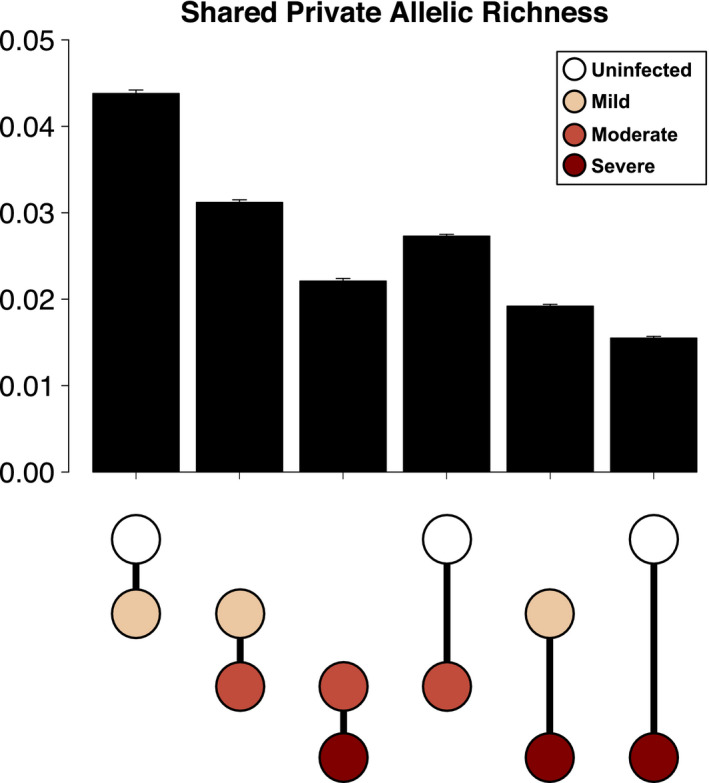
Rarefied private allelic richness shared between mange severity classes

We additionally examined private allelic richness shared between pairwise combinations of infection groups (Figure 3, Table S4). In general, similar groups (where highest mange score was offset by one) shared more unique alleles than disparate groups (where highest mange score was offset by two or three), with the exception of the uninfected–moderate pair. As such, uninfected and mildly infected wolves shared the most alleles (0.0438 ± 0.004), followed by mildly and moderately infected wolves (0.0312 ± 0.003). In contrast, uninfected and severely infected wolves shared the fewest alleles (0.0155 ± 0.0002), with alleles shared by mildly and severely infected wolves similarly low (0.0192 ± 0.002). Within the pairs offset by one mange score, we observed an inverse relationship between infection severity and shared private allele richness. For example, the moderate–severe pair (0.0221 ± 0.0003) shared fewer alleles than both the uninfected–mild (0.0438 ± 0.0004) and mild–moderate (0.0312 ± 0.0003) pairs. This trend also occurred for pairs offset by two mange scores.

### 3.3 Mixed‐effects modeling

**FIGURE 4:**
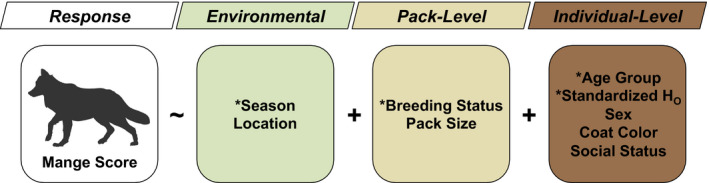
Fixed effects included environmental, pack‐level, and individual‐level variables. Asterisks indicate variables included in the final model. Figure created with BioRender

After constructing our null and global CLMMs, we calculated the variance inflation factor (VIF) for each fixed effect variable included in the saturated model. We observed low collinearity between predictor variables (VIF range 1.18–3.13; Table S5) and initiated our stepwise model reduction procedure. The most parsimonious model contained environmental, pack‐level, and individual‐level variables (Figure 4; Table S6). More specifically, season (*β* = 0.9485, *Z* = 3.166, *p* = .002), breeding status of the pack (*β* = −2.0258, *Z* = −2.584, *p* = .010), and age group (*β* = 1.1769, *Z* = 2.016, *p* = .044) exhibited significant effects, with standardized *H_O_* approaching significance (*β* = −0.8434, *Z* = −1.911, *p* = .056; Table 1). These four variables appeared in all six models with ΔAICc < 2, with all other variables appearing in only one of six top models (Table S6). Parameter estimates for these variables suggested that wolves experienced more severe mange in winter (environment: season) and in nonbreeding packs (pack level: breeding status). Regarding individual‐level variables, adult wolves (age group) and individuals with reduced genetic variation (standardized *H_O_*) also experienced more severe mange. Removal of standardized *H_O_* from the reduced model resulted in significantly worse model fitting (*p* = .041), and subsequent addition of omitted and pairwise interaction terms did not significantly improve AICc (*p* > .05). We therefore retained the reduced model as our most parsimonious CLMM. This model excluded location, pack size, coat color, sex, and social status as significant predictors of mange severity at the individual level.

**TABLE 1:** Parameter estimates (*β*), standard error, *Z*‐score, *p*‐value, and 95% confidence intervals for variables contained in the most parsimonious model predicting mange severity at the individual level

Explanatory variable	*β*	*SE*	*Z*‐score	*p*‐value	CI (2.5%)	CI (97.5%)
Season (winter)	0.9485	0.2996	3.166	.002	0.3612	1.5358
Breeding status (yes)	−2.0258	0.7839	−2.584	.010	−3.5621	−0.4894
Age group (adult)	1.1769	0.5839	2.016	.044	0.0326	2.3213
Standardized *H_O_*	−0.8434	0.4413	−1.911	.056	−1.7083	0.0216

### 3.4 Identifying outlier loci

We identified 410 autosomal sites significantly associated with mange severity after applying BY‐modified FDR correction (*p* < .004). Frequency of the mange‐associated allele was positively associated with mange severity at 224 sites and negatively associated with mange severity at 186 sites. Across all 410 sites, the mange‐associated allele was typically found in the heterozygous state (Table S7). Site annotations included 12 exonic, 171 intronic, 17 near promoters, and 257 intergenic sites, with VEP annotations including 20 low, four moderate, and 847 modifier effects (*N.B.,* many sites had multiple annotations). We identified 42 gene ontological categories that passed the FDR threshold set in *G:PROFILER* (Table S8). Categories included four molecular functions, 16 cellular components, and 22 biological processes (Figure S3a). The majority of categories involved cell barrier function and flexibility (*n* = 11), cell–cell and cell–substrate junctions (*n* = 6), and cell differentiation and development (*n* = 19).

Genic sites queried in the Ensembl, OMIM, and GeneCards databases returned putative functions related to innate and adaptive immunity, autoimmunity and inflammation, cell barriers and adhesion, and skin development and disorders. For example, hematopoietic prostaglandin D synthase (*HPGDS*) has been implicated in the resolution of delayed‐type hypersensitivity responses (Trivedi et al., 2006). Similarly, protein tyrosine phosphatase, nonreceptor type 6 (*PTPN6*) has been linked to heightened inflammation characterized by edema, sustained inflammatory infiltrate, and the delayed wound healing (Lukens et al., 2013). Both loci exhibited decreasing minor allele frequency with increasing infection severity (Figure S3b).

Additional genes were associated with chronic skin disorders, such as psoriasis and peeling skin disease (corneodesmosin, *CDSN*; Matsumoto et al., 2008; Oji et al., 2010), inflamed skin lesions called *hidradenitis suppurativa* (nicastrin, *NCSTN*; Pink et al., 2011), inelastic skin termed *cutis laxa* (Elastin, *ELN*; Hadj‐Rabia et al., 2013), ichthyosis (scaly skin) associated with Refsum disease (peroxisomal biogenesis factor 7, *PEX7*; van den Brink et al., 2003; Schmuth et al., 2013), palmoplantar keratoderma (thickening of the skin around hands and feet) and alopecia (SAM and SH3 domain containing 1, *SASH1*; Courcet et al., 2015), and epithelial cell growth and thickening, termed hyperplasia and hyperkeratosis (SMAD family member 7, *SMAD7*; He et al., 2002). These loci exhibited negative (*ELN*, *SASH1*, and *SMAD7*) and positive (*CDSN*, *NCSTN*, and *PEX7*) relationships between minor allele frequency and infection severity, with no overarching pattern evident in control loci lacking association with mange severity (Figures S3 and S4; Table S9).

### 3.5 Population‐level analyses

**FIGURE 5:**
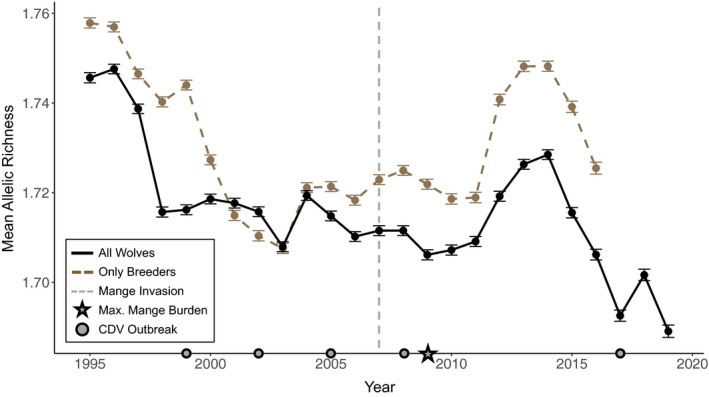
Mean allelic richness rarefied to 20 individuals for all wolves (black solid line) and all known breeders (brown dashed line) alive in each year

We analyzed 76,859 SNPs in 408 unique individuals observed in YNP between 1995 and 2019. Annual datasets ranged from 22 wolves in 2019 to 122 wolves in 2003 (median = 73). Rarefied mean allelic richness decreased through time, with the highest values calculated for 1995 (*A_R_* = 1.7456 ± 0.0011) and 1996 (*A_R_* = 1.7476 ± 0.0011) and the lowest values calculated for 2017 (*A_R_* = 1.6926 ± 0.0012), 2018 (*A_R_* = 1.7017 ± 0.0013), and 2019 (*A_R_* = 1.6891 ± 0.0014; Figure 5). Analysis of breeding individuals was restricted to 1995–2016 due to small sample sizes in 2017–2019. These datasets ranged from 17 wolves in 2016 to 67 wolves in 2003 (median = 41). Although the overall trend was similar, breeding individuals exhibited higher mean allelic richness than the census population in all years except 2001–2003. The highest values were calculated in 1995 (*A_R_* = 1.7579 ± 0.0011) and 1996 (*A_R_* = 1.7570 ± 0.0011), with the lowest values occurring in 2001 (*A_R_* = 1.7149 ± 0.0011), 2002 (*A_R_* = 1.7104 ± 0.0011), and 2003 (*A_R_* = 1.7076 ± 0.0011; Figure 5).

**FIGURE 6:**
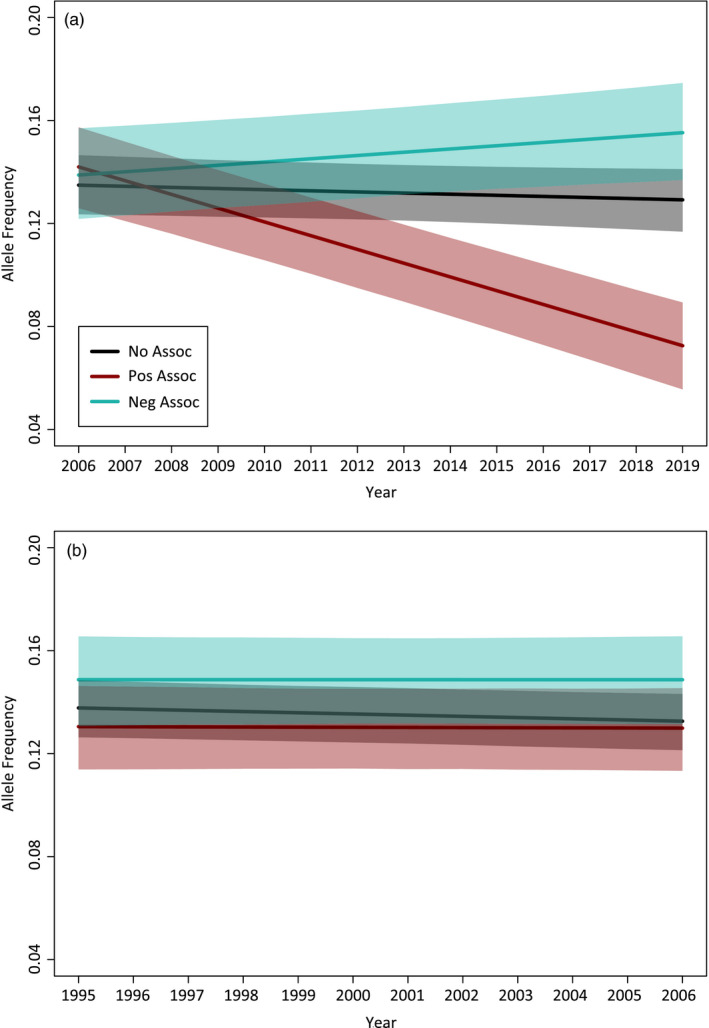
Posterior predictions of the average changes in frequency through time for alleles not associated, positively associated, and negatively associated with mange severity, with 95% credible intervals surrounding the mean. Nonassociated alleles comprise a randomly selected subset of 500 loci. The same analysis was repeated to assess changes in allele frequency (a) after mange invasion of YNP and (b) before mange invasion of YNP

Allele frequency analyses included three bins of loci: (a) no association, (b) positive association, and (c) negative association between the focal allele (typically the minor allele) frequency and mange severity. The majority of mange‐associated loci (*n* = 399/410) had both alleles present in YNP since 1995–1996, with the minor allele emerging for the remaining 11 loci between 1997 and 2003 (Table S10). All mange‐associated alleles were therefore present in the population as standing variation in January 2007, when mange invaded the park. We tested for selection by constructing a mixed‐effects model to estimate whether the average change in allele frequency through the years 2006–2019 depended on mange association. As expected, randomly subsampled nonassociated alleles did not significantly change in frequency on average during this time frame (*β* = 0.003, 95% credible interval [−0.009,0.002]; Figure 6a). Conversely, alleles positively associated with mange severity significantly decreased in frequency on average (*β* = −0.041, 95% credible interval [−0.049, −0.034]; Figure 6a), while alleles negatively associated with mange severity significantly increased in frequency on average (*β* = 0.010, 95% credible interval [0.002,0.018]; Figure 6a). Critically, these significant changes in allele frequency were restricted to the years after mange invaded YNP. From 1995 to 2006, none of these three bins of loci exhibited significant changes in frequency on average (nonassociated, *β* = −0.004, 95% credible interval [−0.007, 0.0001]; positively associated, *β* = 0.000, 95% credible interval [−0.006, 0.005]; negatively associated, *β* = 0.000, 95% credible interval [−0.007, 0.006]; Figure 6b). These results were consistent across four independent subsamples of nonassociated alleles (Figure S5).

## 4 DISCUSSION

In the present study, we characterized the relationship between host genomic variation and disease severity in a wild population of reintroduced canids. Through use of biobanked samples and detailed phenotypic records, we calculated summary statistics of genome‐wide variation and performed a family‐based association study to identify genomic variants linked with mange infection severity. We contextualized genomics within the broad range of factors influencing disease state in YNP, and considered changes in genomic variation through time at the population level. Through these analyses, we found evidence of selection acting on mange‐associated loci following the 2007 invasion of *S. scabiei* mites in YNP. Although numerous studies have catalogued immunogenetics in canids (Aguilar et al., 2004; Arbanasić et al., 2013; Galaverni, Caniglia, Fabbri, Lapalombella, & Randi, 2013; Hedrick, Lee, & Garrigan, 2002; Hedrick, Lee, & Parker, 2000; Kennedy et al., 2011; Marshall, Langille, Hann, & Whitney, 2016), this study was among the first to explore genome‐wide variation within the context of disease severity in a wild canid population. This allowed us to test whether host genomic variation was predictive of disease severity, as suggested by the monoculture effect observed in agricultural settings (Ekroth et al., 2019). Results yielded system‐specific insights, while also contributing to the larger‐scale effort of applying genomic techniques to wildlife disease ecology (Blanchong et al., 2016; DeCandia et al., 2018).

We hypothesized that host genomic variation would predict mange infection severity rather than susceptibility, given the mode of transmission and pathology of sarcoptic mange. Transmission of *S. scabiei* mites occurs upon contact with an infected individual or fomite, such as a mange‐infected den (Montecino‐Latorre et al., 2019; Pence & Ueckermann, 2002). Presumably, exposed wolves have an equal probability of infection regardless of their genomic diversity. Inter‐individual differences subsequently emerge due to the immune response mounted by the host (Nimmervoll et al., 2013; Oleaga et al., 2012), which is likely to be under genetic control (Steinke, Borish, & Rosenwasser, 2003). In the present study, we observed an inverse relationship between host genomic variation and mange infection severity in YNP wolves. This supports the paradigm that genetic variation plays an important role in wildlife disease (King & Lively, 2012; Lively, 2010; Luong, Heath, & Polak, 2007; Spielman, Brook, Briscoe, et al., 2004). Additional evidence includes heterozygous house finches (*Carpodacus mexicanus*) that exhibited reduced disease severity and mounted stronger immune responses than homozygous finches after experimental inoculation with *Mycoplasma gallisepticum* (Hawley, Sydenstricker, Kollias, & Dhondt, 2005). Similarly, outbred guppies (*Poecilia reticulata*) exhibited lower *Gyrodactylus turnbulli* parasite intensities and shorter infection durations when compared to inbred individuals (Smallbone, Oosterhout, & Cable, 2016). Here, YNP wolves exhibiting mild mange symptoms possessed higher levels of genome‐wide variation than wolves exhibiting more severe symptoms.

Patterns of genome‐wide variation suggested that host diversity was an important predictor of mange severity in YNP wolves. However, wildlife disease dynamics are known to be impacted by host environment, demography, and behavior, as well (Ezenwa et al., 2016; Parratt, Numminen, & Laine, 2016; Silk et al., 2019). We therefore used mixed‐effects modeling to quantitatively assess the role of genetic diversity in shaping the landscape of mange infection severity at the individual level. The most parsimonious model highlighted the multifactorial nature of disease state in wildlife through inclusion of genetic (standardized *H_O_*), environmental (season), and demographic (breeding status of the pack and individual age) variables. Critically, we found model support for an inverse relationship between genetic diversity and mange severity. This result confirmed the relevance of genome‐wide variation in predicting mange severity in YNP wolves alongside environmental, behavioral, and demographic factors.

To build upon this result and capture the complex nature of disease, we explored other model relationships associated with mange severity. Regarding season, we found evidence that wolves presented more severe mange in winter, when cold ambient temperatures render thermoregulation more difficult. This result supports previous studies examining mange prevalence (Almberg et al., 2012), mortality risk (Almberg et al., 2015), and energetics (Cross et al., 2016) in YNP wolves and other species impacted by mange (Martin et al., 2018). Season may also contribute to our finding that nonbreeding packs were more likely to present severe mange. Breeding season for YNP wolves (mid‐February mating; mid‐April birth) directly follows the mean severity window for infected packs (September 2–February 2; Almberg et al., 2015). Mangy individuals may exhibit poor body condition during breeding season, reducing breeding likelihood and efficacy (Stahler et al., 2013), as seen in other host–parasite systems (Holand et al., 2015; Marzal, De Lope, Navarro, & Møller, 2005; Møller, 2002; Sarasa et al., 2011). Poor body condition may also be influenced by age, as adult wolves exhibited worse mange than yearlings. This finding is consistent with reports for age/mange relationships in Iberian wolves and coyotes (Oleaga et al., 2011; Pence et al., 1983), and divergent from reports in red foxes and dogs (Fazal et al., 2014; Feather et al., 2010; Newman, Baker, & Harris, 2002). These differences in the literature, and our study in particular, may result from the demographics of the dataset. For example, we excluded any wolf that had fewer than three observations, which may have systematically excluded fatal mange infections in pups, as seen in the Silver pack in 2010 (Smith et al., 2011). We therefore recommend further study of age‐specific outcomes with mange infections in both wolves and canids, more broadly.

Following our findings that genome‐wide variation significantly predicts mange severity, we discovered specific loci associated with the highest mange score recorded per wolf. These loci were found in genes related to innate and adaptive immunity, autoimmunity and inflammation, cell barriers and adhesion, and skin development and disorders. For example, reduced minor allele frequency was associated with severe mange in genes *HPGDS* and *PTPN6,* which have been previously linked to immunopathology, inflammation, and delayed wound healing in mice (Lukens et al., 2013; Trivedi et al., 2006). Additional loci were discovered in genes related to chronic skin conditions. Associated genes included *CDSN* (psoriasis and peeling skin disease; Matsumoto et al., 2008; Oji et al., 2010), *NCSTN* (*hidradenitis suppurativa*; Pink et al., 2011), *ELN* (*cutis laxa;* Hadj‐Rabia et al., 2013), *PEX7* (ichthyosis; van den Brink et al., 2003; Schmuth et al., 2013), *SASH1* (palmoplantar keratoderma and alopecia; Courcet et al., 2015), and *SMAD7* (hyperplasia and hyperkeratosis; He et al., 2002). These annotations and skin conditions matched symptoms of severe mange across host taxa (Almberg et al., 2012; Little et al., 1998; Niedringhaus, Brown, Sweeley, & Yabsley, 2019; Oleaga et al., 2012; Pence & Ueckermann, 2002), and were consistent with inflammation‐induced immunopathology and hypersensitivity presented in allergic and autoimmune disorders (Barker, 2001; Barnes, 2010; Bin & Leung, 2016; Esaki et al., 2015; Liang, Chang, & Lu, 2016; Nattkemper et al., 2018; Quraishi et al., 2015; Rodríguez et al., 2014; Sonkoly et al., 2010). While it is possible that mange‐associated alleles may also influence individual‐level risk during CDV outbreaks, annotations more closely matched the pathology of mange. Overall, these results mirror other wildlife studies that uncovered disease‐relevant genes in diverse host–parasite systems (Batley et al., 2019; Donaldson et al., 2017; Elbers et al., 2018; Ellison et al., 2014; Margres et al., 2018).

The relevance of host genomics to disease risk is not restricted to the individual level. Many of these processes scale to inform population‐level dynamics through time. In YNP wolves, temporal analyses confirmed our hypothesis that genomic variation has decreased since the initial reintroduction event, despite ephemeral fluctuations. Although some fluctuations coincide with CDV outbreaks, the inconsistent pattern suggests that disease was not the primary driver of long‐term decreases in genome‐wide variation. Instead, this pattern may result from YNP’s status as a source population for the surrounding Greater Yellowstone Ecosystem, as few wolves successfully disperse into the park (vonHoldt et al., 2010; vonHoldt et al., 2008). Notably, the mating structure of wolves precludes all individuals from reproducing (Mech & Boitani, 2003), thereby reducing the effective population size (vonHoldt et al., 2008). While the overall pattern of diversity was consistent between all wolves and known breeders, reproductive individuals exhibited higher levels of genome‐wide variation in all years except 2001–2003. This upward shift may slow the pace of genomic diversity loss through time, although further study is needed on mate choice and its long‐term effects on variation in YNP.

**FIGURE 7:**
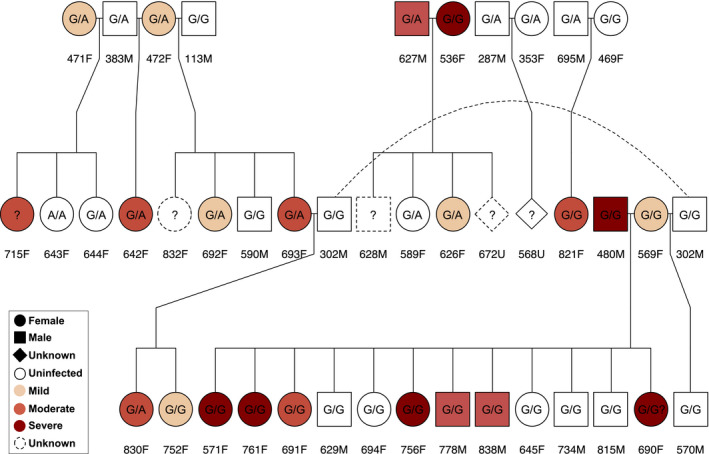
Sarcoptic mange was implicated in the dissolution of the Druid Peak pack in late 2009 and early 2010, when numerous pack members became infected. This pedigree contains a subset of Druid wolves shaded to indicate mange infection severity. Genotype at the mange‐associated locus contained in gene *PTPN6* is indicated, when available, to illustrate how family‐based association links genotypes with phenotypes while controlling for relatedness. Similar analyses were conducted for all loci analyzed by *GEMMA*. Dashed lines connect the same individual to parentage events occurring in different parts of the pedigree

Decline in genome‐wide variation at the population level may increase the prevalence of severe mange infections in the future, given the inverse relationship observed at the genomic scale. While higher levels of diversity maintained by breeders may ameliorate this risk, mange can still negatively affect YNP wolves. For example, mange has been implicated in the dissolution of previously stable packs, such as the Druid Peak pack (Figure 7; Almberg et al., 2012). Although two litters were born in April 2009, Druid wolves began to exhibit symptoms of mange infection soon after. By the end of October, the pack had lost alpha female 569F to intraspecific conflict, numerous wolves to dispersal or death, and all pups to mange or its associated symptoms (Smith et al., 2009). Surviving members fragmented into smaller groups in early 2010, and the exceptionally long tenure of the Druid Peak pack was over by year's end (Smith et al., 2011). Similar stories emerged from the Leopold, Everts, and Silver packs (Almberg et al., 2012Yellowstone National Park Wolf Project Annual; Smith et al., 2011), emphasizing the scaling effects of mange infection on individuals, packs, and the greater YNP wolf population.

As exemplified by the Druid Peak case study, mange‐mediated mortality and pack dissolution can impose strong selective pressures on YNP wolves. Consistent with our expectations, analyses of allele frequency through time revealed signatures of selection acting on mange‐associated loci. Similar effects have been seen in response to *Mycoplasma galliseptum* (Bonneaud et al., 2011), devil facial tumor disease (Epstein et al., 2016), and chytridiomycosis (Savage & Zamudio, 2016). In the present study, we observed significant reductions in the average frequency of alleles positively associated with mange severity between 2006 and 2019 (after mange invaded the park), with no change evident between 1995 and 2006. We additionally observed evidence of selection increasing alleles negatively associated with mange severity between 2006 and 2019; however, credible intervals overlapped with the nonassociated group, which exhibited no change between 1995 and 2006 and between 2006 and 2019. Considered together, these results suggested that there are stronger selective pressures acting to remove alleles associated with severe mange (i.e., positively associated alleles) than to increase the frequency of alleles associated with mild mange (i.e., negatively associated alleles).

The observed relationship between host genomic variation and disease severity in YNP wolves at the individual and population levels highlights the relevance of molecular variation to wildlife populations (Frankham, 2003). This is particularly important for host species threatened by disease, whether through epizootic outbreaks or the slow invasion of enzootics (Daszak, Cunningham, & Hyatt, 2000). These results support the paradigm that host genomic variation can buffer against disease risk, as seen in agriculture's monoculture effect. They further emphasize the importance of considering genome‐wide variation and disease‐relevant loci when studying host–parasite dynamics, particularly in longer‐evolved systems. Using YNP wolves as an example, declines in genome‐wide variation through time may increase the likelihood of severe mange infections. However, removal of harmful mange‐associated alleles may counteract that risk. Monitoring summary metrics of diversity alongside disease‐associated loci will enable more accurate risk assessment and outbreak predictions, although YNP wolves are not currently treated or vaccinated against disease. In more heavily managed wildlife systems, similar analyses can directly inform conservation action. While further studies are needed to assess the universality of these trends, we posit that the maintenance of genetic variation should remain a priority during founder selection, reintroduction, and subsequent population management of at‐risk populations. For species harboring inbred genomes, we further recommend exploration of additional molecular mechanisms that may influence disease risk in the absence of genomic variation (such as gene regulation or the host‐associated microbiome). The integration of molecular and disease ecologies presents a powerful opportunity to elucidate the factors underlying disease risk, as well as the evolutionary effects of disease on wildlife. These insights can then inform best practices for disease management and wildlife conservation.

## CONFLICT OF INTEREST

None declared.

## Supporting information

Appendix S1

## Data Availability

Mapped bam files for the samples included in this study are available on NCBI’s public Sequence Read Archive under BioProjects PRJNA577957 and PRJNA660734. Please see supplementary information for metadata and BioSample Accession Numbers.
