# Supplementary material for: Sarcoptic mange severity is associated with reduced genomic variation and evidence of selection in Yellowstone National Park wolves (Canis lupus)
Source: Evol Appl. 2020 Sep 20;14(2):429–45. doi: 10.1111/eva.13127 (PMC7896714; doi:10.1111/eva.13127)
Supplement: Supplementary file 1 — Appendix S1 [file EVA-14-429-s001.zip › eva13127-sup-0001-AppendixS1.docx]

**Table S1.** Hypotheses underlying each fixed effect variable considered during model selection. Variables included season (winter/summer), location (northern range/interior), breeding status of the pack (yes/no), pack size (range: 1-18), sex (male/female), coat color (gray/black), age group (yearling/adult), social status (subordinate/alpha), and standardized observed heterozygosity (H_O_ range: -3.6439-1.7393).

| **Fixed Effect** | **Hypothesis** | **Rationale** | **Sources** |
| --- | --- | --- | --- |
| Season | more severe in winter | thermoregulation difficult; mange more prevalent | Almberg et al. 2015; Cross et al. 2016 |
| Location | more severe in northern range | higher wolf density; mange more prevalent | Almberg et al. 2012 |
| BreedingStatus | more severe in non-breeding packs | severely inf. wolves less likely to breed | Stahler et al. 2013 |
| PackSize | more severe in larger packs | severely inf. wolves may survive longer in larger packs | Almberg et al. 2015 |
| Sex | no effect | sex does not predict mange severity in dogs | Feather et al. 2010; Fazal et al. 2014 |
| CoatColor | more severe with gray coat color | k-locus associated with immunity; higher risk of mange-related mortality with gray coat color | Candille et al. 2007; Almberg et al. 2015 |
| AgeGroup | more severe in adult wolves | older wolves and coyotes had more severe mange than younger individuals | Pence et al. 1983; Oleaga et al. 2011 |
| SocialStatus | more severe in subordinate | alphas may lose social status with severe infection | Mech and Boitani 2003 |
| ObsHetStd | more severe with lower H_O_ | theory predicts heightened disease risk with decreased genetic diversity | Spielman et al. 2004; DeCandia et al. 2018 |

**Fig. S1.** Mange score at the time of observation for 117 wolves exposed to mange. Figure adapted from Almberg et al. 2012.

**Table S2.** Genetic diversity statistics calculated across 76,859 loci genotyped in 117 wolves grouped by infection status (uninfected vs. infected) and severity (highest mange score 0, 1, 2, and 3). Metrics include: sample size (N), percentage polymorphic loci (%Poly), number of private alleles (PAS), observed heterozygosity (H_O_), expected heterozygosity (H_E_), minor allele frequency (MAF), and nucleotide diversity (π). Standard error (SE) is given in parentheses.

| **Mange Status (Highest Score)** | **N** | **%Poly** | **PAS** | **H_O_ (SE)** | **H_E_ (SE)** | **MAF (SE)** | **π (SE)** |
| --- | --- | --- | --- | --- | --- | --- | --- |
| Uninfected (0) | 49 | 96.1618 | 2242 | 0.1942 (0.0006) | 0.1856 (0.0005) | 0.1239 (0.0005) | 0.1876 (0.0006) |
| Infected (1, 2, 3) | 68 | 97.0830 | 2950 | 0.1986 (0.0006) | 0.1874 (0.0005) | 0.1249 (0.0005) | 0.1888 (0.0005) |
| Mild (1) | 29 | 91.0967 | 455 | 0.2039 (0.0006) | 0.1922 (0.0006) | 0.1296 (0.0005) | 0.1957 (0.0006) |
| Moderate (2) | 26 | 86.7263 | 227 | 0.1950 (0.0007) | 0.1804 (0.0006) | 0.1219 (0.0005) | 0.1843 (0.0006) |
| Severe (3) | 13 | 66.9941 | 56 | 0.1943 (0.0008) | 0.1697 (0.0006) | 0.1203 (0.0005) | 0.1769 (0.0006) |

**Table S3.** Rarefied allelic diversity metrics comparing wolves grouped by infection status (uninfected vs. infected) and severity (highest mange score 0, 1, 2, and 3). Variables include the number of loci analyzed (Loci), rarefied sample size (g), mean allelic richness (A_R_), and mean private allelic richness (PA_R_), with standard error (SE) given in parentheses.

| **Mange Status (Highest Score)** | **Loci** | **g** | **A_R_ (SE)** | **PA_R_ (SE)** |
| --- | --- | --- | --- | --- |
| Uninfected (0) | 76,859 | 78 | 1.9431 (0.0007) | 0.0653 (0.0007) |
| Infected (1, 2, 3) | 76,859 | 78 | 1.9276 (0.0007) | 0.0498 (0.0006) |
| Uninfected (0) | 76,475 | 20 | 1.7190 (0.0011) | 0.0469 (0.0004) |
| Mild (1) | 76,475 | 20 | 1.7360 (0.0011) | 0.0425 (0.0004) |
| Moderate (2) | 76,475 | 20 | 1.7059 (0.0012) | 0.0323 (0.0003) |
| Severe (3) | 76,475 | 20 | 1.6347 (0.0016) | 0.0195 (0.0003) |

**Fig. S2.** Genetic diversity statistics for mange infected wolves grouped by uninfected (highest mange score 0), mild (highest mange score 1), moderate (highest mange score 2), and severe (highest mange score 3) infection severity. Metrics include: (**A**) percentage polymorphic loci, (**B**) number of private alleles, (**C**) observed heterozygosity, (**D**) expected heterozygosity, (**E**) minor allele frequency, (**F**) nucleotide diversity, (**G**) rarefied mean allelic richness, and (**H**) rarefied mean private allelic richness. **Table S4.** Rarefied private allelic richness shared between infection severity groups. Variables include the number of loci analyzed (Loci), rarefied sample size (g), and mean shared private allelic richness (Shared PA_R_), with standard error (SE) given in parentheses.

| **Mange Status - Pair** | **Loci** | **g** | **Shared PA_R_ (SE)** |
| --- | --- | --- | --- |
| Uninfected - Mild | 76,475 | 20 | 0.0438 (0.0004) |
| Uninfected - Moderate | 76,475 | 20 | 0.0273 (0.0002) |
| Uninfected - Severe | 76,475 | 20 | 0.0155 (0.0002) |
| Mild - Moderate | 76,475 | 20 | 0.0312 (0.0003) |
| Mild – Severe | 76,475 | 20 | 0.0192 (0.0002) |
| Moderate - Severe | 76,475 | 20 | 0.0221 (0.0003) |

**Table S5.** Variance inflation factor (VIF) and increased standard error (SE) suggest low collinearity between variables.

| **Parameter** | **VIF** | **Increased SE** |
| --- | --- | --- |
| Season | 1.18 | 1.09 |
| Location | 3.13 | 1.77 |
| BreedingStatus | 1.98 | 1.41 |
| PackSize | 2.53 | 1.59 |
| Sex | 1.37 | 1.17 |
| CoatColor | 1.35 | 1.16 |
| AgeGroup | 1.51 | 1.23 |
| SocialStatus | 1.29 | 1.14 |
| ObsHetStd | 1.36 | 1.17 |

**Table S6.** Model formula, corrected delta Akaike Information Criterion adjusted for small sample size (ΔAICc), and weighted AICc calculated for cumulative link mixed models tested during stepwise model reduction. Mange score at the time of observation served as the response variable. Random effects in all models included: individual identifier, pack membership, and year observed. The global model contained all nine fixed effects. Environmental variables included: observation season (*Season*) and location in the park (*Location*). Pack-level variables included: breeding status (*BreedingStatus*) and size (*PackSize*) of the pack. Individual-level variables included: sex (*Sex*), coat color (*CoatColor*), age group (*AgeGroup*), social status (*SocialStatus*), and standardized observed heterozygosity (*ObsHetStd*).

| **Model Formula** | **ΔAICc** | **AICc *wi*** |
| --- | --- | --- |
| ~ Season + BreedingStatus + AgeGroup + ObsHetStd | 0.000 | 0.231 |
| ~ Season + BreedingStatus + AgeGroup + ObsHetStd + CoatColor | 1.091 | 0.134 |
| ~ Season + BreedingStatus + AgeGroup + ObsHetStd + PackSize | 1.136 | 0.131 |
| ~ Season + BreedingStatus + AgeGroup + ObsHetStd + Location | 1.590 | 0.104 |
| ~ Season + BreedingStatus + AgeGroup + ObsHetStd + Sex | 1.590 | 0.104 |
| ~ Season + BreedingStatus + AgeGroup + ObsHetStd + SocialStatus | 1.829 | 0.092 |
| ~ Season + BreedingStatus + AgeGroup | 2.095 | 0.081 |
| ~ Season + BreedingStatus + PackSize + CoatColor + AgeGroup + ObsHetStd | 2.311 | 0.073 |
| ~ Season + BreedingStatus + PackSize + Sex + CoatColor + AgeGroup + ObsHetStd | 3.946 | 0.032 |
| ~ Season + BreedingStatus + PackSize + Sex + CoatColor + AgeGroup + SocialStatus + ObsHetStd | 5.669 | 0.014 |
| ~ Season + Location + BreedingStatus + PackSize + Sex + CoatColor + AgeGroup + SocialStatus + ObsHetStd | 7.470 | 0.006 |
| ~ 1 | 15.911 | 0.000 |
| *Random effects: individual identifier, pack membership, and year observed* |  |  |

**Table S7.** Genotype counts and proportions at 410 loci significantly associated with mange severity. The mange-associated allele (coded 1) was positively associated with infection severity at 224 loci, and negatively associated with infection severity at 186 loci. The majority of mange-associated alleles were present in the heterozygous (0/1) rather than homozygous (1/1) state.

|  |  | | **Positively Associated Loci (n=224)** | | | | | | **Negatively Associated Loci (n=186)** | | | | | |  |
| --- | --- | --- | --- | --- | --- | --- | --- | --- | --- | --- | --- | --- | --- | --- | --- |
|  |  | | **Genotype Counts** | | | **Genotype Proportions** | | | **Genotype Counts** | | | **Genotype Proportions** | | |  |
| **Mange Severity** | | | **N** | **0/0** | **0/1** | **1/1** | **0/0** | **0/1** | **1/1** | **0/0** | **0/1** | **1/1** | **0/0** | **0/1** | **1/1** |
| Uninfected | | | 49 | 9242 | 1182 | 97 | 0.8784 | 0.1123 | 0.0092 | 5817 | 2483 | 436 | 0.6659 | 0.2842 | 0.0499 |
| Mild | | | 29 | 5150 | 1059 | 164 | 0.8081 | 0.1662 | 0.0257 | 3930 | 1171 | 166 | 0.7462 | 0.2223 | 0.0315 |
| Moderate | | | 26 | 3905 | 1352 | 196 | 0.7161 | 0.2479 | 0.0359 | 3684 | 694 | 58 | 0.8305 | 0.1564 | 0.0131 |
| Severe | | | 13 | 1578 | 1057 | 158 | 0.5650 | 0.3784 | 0.0566 | 2047 | 257 | 7 | 0.8858 | 0.1112 | 0.0030 |
| *All Exposed* | | | *117* | *19875* | *4650* | *615* | *0.7906* | *0.1850* | *0.0245* | *15478* | *4605* | *667* | *0.7459* | *0.2219* | *0.0321* |

**Table S8.** Gene ontology categories significantly enriched within the queried gene set. Columns include data source (MF=molecular function, BP=biological process, and CC=cellular component), term name, term identifier, adjusted *p*-value, and intersection of sites with that annotation.

| **Source** | **Term Name** | **Term ID** | **Adjusted *p*** | **Sites** |
| --- | --- | --- | --- | --- |
| MF | vinculin binding | GO:0017166 | 0.0158 | 3 |
| MF | actinin binding | GO:0042805 | 0.0286 | 4 |
| MF | cytoskeletal protein binding | GO:0008092 | 0.0497 | 16 |
| MF | voltage-gated calcium channel activity involved in positive regulation of presynaptic cytosolic calcium levels | GO:0099635 | 0.0497 | 2 |
| CC | cell-cell junction | GO:0005911 | 0.0072 | 10 |
| CC | intercalated disc | GO:0014704 | 0.0339 | 3 |
| CC | cell junction | GO:0030054 | 0.0334 | 12 |
| CC | anchoring junction | GO:0070161 | 0.0164 | 11 |
| CC | stress fiber | GO:0001725 | 0.0009 | 6 |
| CC | actin filament bundle | GO:0032432 | 0.0010 | 6 |
| CC | actomyosin | GO:0042641 | 0.0010 | 6 |
| CC | contractile actin filament bundle | GO:0097517 | 0.0009 | 6 |
| CC | phagocytic cup | GO:0001891 | 0.0072 | 3 |
| CC | plasma membrane | GO:0005886 | 0.0072 | 48 |
| CC | sarcolemma | GO:0042383 | 0.0125 | 5 |
| CC | cell periphery | GO:0071944 | 0.0056 | 50 |
| CC | plasma membrane bounded cell projection | GO:0120025 | 0.0390 | 21 |
| CC | cell projection | GO:0042995 | 0.0300 | 22 |
| CC | axon | GO:0030424 | 0.0334 | 9 |
| CC | L-type voltage-gated calcium channel complex | GO:1990454 | 0.0334 | 2 |
| BP | multicellular organismal process | GO:0032501 | 0.0279 | 63 |
| BP | cell projection organization | GO:0030030 | 0.0061 | 23 |
| BP | plasma membrane bounded cell projection organization | GO:0120036 | 0.0061 | 23 |
| BP | cell morphogenesis | GO:0000902 | 0.0213 | 18 |
| BP | multicellular organism development | GO:0007275 | 0.0279 | 47 |
| BP | nervous system development | GO:0007399 | 0.0423 | 26 |
| BP | anatomical structure morphogenesis | GO:0009653 | 0.0061 | 34 |
| BP | neurogenesis | GO:0022008 | 0.0168 | 23 |
| BP | developmental process | GO:0032502 | 0.0061 | 58 |
| BP | cellular component morphogenesis | GO:0032989 | 0.0061 | 21 |
| BP | cell development | GO:0048468 | 0.0061 | 29 |
| BP | system development | GO:0048731 | 0.0291 | 44 |
| BP | anatomical structure development | GO:0048856 | 0.0215 | 52 |
| BP | cellular developmental process | GO:0048869 | 0.0183 | 42 |
| BP | negative regulation of chondrocyte proliferation | GO:1902731 | 0.0471 | 2 |
| BP | neuron projection development | GO:0031175 | 0.0256 | 16 |
| BP | neuron projection morphogenesis | GO:0048812 | 0.0471 | 12 |

| BP | cell morphogenesis involved in differentiation | GO:0000904 | 0.0279 | 14 |
| --- | --- | --- | --- | --- |
| BP | cell differentiation | GO:0030154 | 0.0061 | 42 |
| BP | neuron differentiation | GO:0030182 | 0.0370 | 19 |
| BP | neuron development | GO:0048666 | 0.0279 | 17 |
| BP | generation of neurons | GO:0048699 | 0.0279 | 21 |

**­­**

**Figure S3.** (**A**) Gene ontological analyses revealed 42 significantly enriched categories, including four molecular functions (MF), 16 cellular components (CC), and 22 biological processes (BP). The frequency of the mange-associated allele exhibited both **(B**) negative and (**C**) positive associations with mange severity. Loci of interest included: PTPN6 (P6), HPGDS (H), SASH1 (S1), SMAD7 (S7), ELN (E), CDSN (C), NCSTN (N), and PEX7 (P7).

**Figure S4.** Minor allele frequency by mange severity class for eight non-associated loci.

**Table S9.** Gene identifier (GeneID), chromosome (chr), position (pos), minor allele (MinAllele), major allele (MajAllele), beta (*β*), standard error (SE), and *p*-value for eight loci significantly associated with highest mange score, and eight loci with no significant association with mange infection severity.

| **GeneID** | **Chr** | **Pos** | **MinAllele** | **MajAllele** | ***β*** | **SE** | ***p*-value** |
| --- | --- | --- | --- | --- | --- | --- | --- |
| PEX7 | 1 | 29338093 | A | C | 1.7203 | 0.4604 | <0.0001 |
| SASH1 | 1 | 39314565 | G | C | -0.8652 | 0.2800 | 0.0021 |
| ELN | 6 | 6319836 | T | C | -1.2146 | 0.4529 | 0.0033 |
| SMAD7 | 7 | 79723406 | T | C | -0.5872 | 0.2342 | 0.0028 |
| CDSN | 12 | 818496 | A | G | 0.5819 | 0.2463 | 0.0040 |
| PTPN6 | 27 | 38096413 | A | G | -0.5920 | 0.1867 | 0.0037 |
| HPGDS | 32 | 17168778 | T | C | -0.5713 | 0.2191 | 0.0015 |
| NCSTN | 38 | 21844278 | T | G | 0.9281 | 0.2922 | 0.0022 |
| *-* | 5 | 17584777 | G | A | -0.0372 | 0.4219 | 1.0000 |
| *-* | 5 | 50645197 | T | C | 0.0519 | 0.2759 | 1.0000 |
| *-* | 8 | 64863777 | T | C | -0.0303 | 0.2696 | 1.0000 |
| *-* | 12 | 72313523 | A | G | -0.1339 | 0.4512 | 1.0000 |
| *-* | 23 | 35866785 | G | A | 0.0755 | 0.1854 | 1.0000 |
| *-* | 25 | 43406159 | G | A | 0.1274 | 0.2987 | 1.0000 |
| *-* | 26 | 12209367 | T | C | -0.0037 | 0.1591 | 1.0000 |
| *-* | 27 | 7984903 | C | G | -0.0511 | 0.1569 | 1.0000 |

**Table S10.** Number of mange-associated loci (n=410 total) with two alleles present (2 Alleles) and one allele present (1 Allele) between 1995-2003. The majority of loci exhibited both alleles in founder wolves (1995-1996). Minor alleles for the remaining 11 loci emerged between 1997-2003.

|  | ***Mange-Associated Loci*** | |
| --- | --- | --- |
| **Year** | **2 Alleles** | **1 Allele** |
| 1995 | 396 | 14 |
| 1996 | 399 | 11 |
| 1997 | 404 | 6 |
| 1998 | 406 | 4 |
| 1999 | 407 | 3 |
| 2000 | 409 | 1 |
| 2001 | 409 | 1 |
| 2002 | 409 | 1 |
| 2003 | 410 | 0 |
|  |  |  |

**Figure S5.** Posterior predictions of the average changes in frequency through time for alleles not associated, positively associated, and negatively associated with mange severity, with 95% credible intervals around the mean. Non-associated alleles comprise randomly selected subsets of 500 loci. These are three replicates (**A** and **B**, **C** and **D**, **E** and **F**) of the same analysis presented in the main text. Like the analysis in the main text, each of these replicates assesses changes in allele frequency (**A**, **C**, **E**) after mange invasion of YNP and (**B**, **D**, **F**) before mange invasion of YNP.
